# Tree of Life Synagogue Shooting in Pittsburgh: Preparedness, Prehospital Care, and Lessons Learned

**DOI:** 10.5811/westjem.2019.11.42809

**Published:** 2020-02-21

**Authors:** Adam Z. Tobias, Ronald N. Roth, Leonard S. Weiss, Keith Murray, Donald M. Yealy

**Affiliations:** * University of Pittsburgh School of Medicine, Department of Emergency Medicine, Pittsburgh, Pennsylvania; † Allegheny Health Network, Department of Emergency Medicine, Pittsburgh, Pennsylvania

## Abstract

On Saturday, October 27, 2018, a man with anti-Semitic motivations entered Tree of Life synagogue in the Squirrel Hill section of Pittsburgh, Pennsylvania; he had an AR-15 semi-automatic rifle and three handguns, opening fire upon worshippers. Eventually 11 civilians died at the scene and eight people sustained non-fatal injuries, including five police officers. Each person injured but alive at the scene received care at one of three local level-one trauma centers. The injured had wounds often seen in war-settings, with the signature of high velocity weaponry. We describe the scene response, specific elements of our hospital plans, the overall out-of-hospital preparedness in Pittsburgh, and the lessons learned.

## INTRODUCTION

On Saturday, October 27, 2018, a man with anti-Semitic motivations entered Tree of Life synagogue in the Squirrel Hill section of Pittsburgh, Pennsylvania. He had an AR-15 semi-automatic rifle and three handguns, and used these to open fire upon the worshippers. Eventually, eleven civilians died at the scene and eight people, including five police officers, sustained non-fatal injuries. Each person injured but alive at the scene received care at one of three local level-one trauma centers and survived. We describe elements of out-of-hospital and in-hospital preparedness in Pittsburgh, medical response during the event, and the lessons learned.

## PREPAREDNESS IN PITTSBURGH

### Prehospital Physician Response

Since its origin in 1981, the Emergency Medicine (EM) residency at the University of Pittsburgh includes a “24/7/365” physician response to specific out of hospital emergencies.^1^,^2^ Emergency Medicine residents (PGY II and PGY III) with Emergency Medical Services (EMS) training and certification staff a response vehicle equipped with radios, emergency warning lights, medications, and medical equipment; they are overseen by a certified attending EMS medical command physician available by radio and phone. Emergency medicine faculty and residents work closely with members of the City of Pittsburgh Bureau of Public Safety (Police, Fire, and EMS) to give protocol and on-scene medical direction while gaining out of hospital care experience.

### Tactical EMS in Pittsburgh

The National Tactical Officers Association recommends that special weapons and tactics (SWAT) teams include trained tactical emergency medical providers.^3^ In 2011 the City of Pittsburgh EMS division enrolled 16 paramedics into SWAT courses and subsequently integrated them into a Tactical EMS (TEMS team). Members of the TEMS team assist with medical threat assessment, pre-deployment team preventative health care, and point-of-injury medical care. The SWAT/TEMS Regional Medical Director, an emergency physician serving as a faculty member in the residency training program at the University of Pittsburgh, trains and deploys with the team. Each SWAT operator carries a standardized Individual First Aid Kit (IFAK) and tourniquet (Table 1), and the TEMS medics carry similar equipment augmented with additional medical gear.

### Out-of-Hospital Preparation and Practice

Based on learning from prior events, UPMC and the City of Pittsburgh recognized the importance of joint training and response coordinated centrally across all arms of local public safety. In 2017, the city began a joint active shooter training series for public safety agencies, beginning with a formalized introductory course, followed by focused agency level training, and finally, ongoing joint exercises of varying scales. The goal was to develop and practice a preferred model of response to active shooter events that would function in parallel with tactical operations of SWAT and TEMS units.

The joint model utilizes combined response teams that allow first responders rapid access to patients to provide immediate lifesaving interventions, rapid extrication, and transport to a trauma facility under the protection of law enforcement personnel. Formation of a Rescue Task Force (RTF) made up of SWAT and TEMS personnel aids in early patient intervention beyond the cold zone and with limited staging delay.

On-scene medical preparation for law enforcement and first responders is led by the City Bureau of EMS, with a focus on standardizing self- and buddy-care of law enforcement personnel along with immediate external hemorrhage control of victims by any available responder. Tourniquet training and dissemination, along with adding IFAKs to duty gear, followed in all public safety agencies. The City also added protective ballistic body armor to standard EMS uniforms.

Our EMS providers are trained in systematic triage and implementation of life-saving interventions in zones of active fire, upon extrication from danger and during transport.

This training uses the Sort, Assess, Lifesaving Interventions, Treatment/Transport (SALT) triage system and the principles of Tactical Combat Casualty Care, teaching techniques for extremity and junctional hemorrhage control and utilization of hemostatic agents. The program also focuses on key rapid life-saving interventions, including airway maneuvers and ventilation, chest decompression and seals, intraosseous access, fluid resuscitation principles, and optimal care of the head injury patient. This background translated into efficient and directed care on scene at Tree of Life.

Years before this event, the Pittsburgh Bureau of Public Safety proactively developed an active threat plan and practiced in advance; they did this after heeding experiences locally and nationally. The most recent preparedness session occurred just months earlier, a few blocks from the synagogue involved.

### In-Hospital Preparation

University of Pittsburgh Medical Center Presbyterian University Hospital, which received the bulk of the patients from this event, has a Mass Casualty Incident(MCI) plan refined through several years of exercises and informed by lessons learned elsewhere, especially in Israel. The approach uses the underlying principle of keeping care as simple as possible.^4^ Based on the Israeli model, “Job-Action cards” exist (e.g., ED attending, charge nurse, triage nurse, lead trauma attending, etc.) and are distributed at the time of MCI plan activation. Each card is a single laminated page with a stepwise checklist of actions to be completed. Provider instructions are simple and focus on simplicity of duties and roles: “You don’t need to memorize the plan, you just have to know where to find it when you need it and then follow the checklist.”

The plan calls for a lead ED attending and trauma surgeon to divide providers into care teams. The anesthesia liaison in the ED coordinates with the operating rooms to cancel elective surgery. The critical care liaison works with the Administrator on Duty (AOD) to create Intensive Care Unit capacity. The internal medicine liaison prepares to call in additional inpatient staff and create capacity.

The hospital central supply unit sends three large pre-prepared disaster carts with additional trauma and respiratory supplies. These carts are kept centrally and sent to the ED with MCI plan activation. Similarly, the hospital pharmacy maintains an emergency cache of medications for pain and rapid sequence induction which are also automatically sent to the ED. The blood bank sends a pre-prepared cache of emergency blood products.

### Community Readiness

Over the past three years, our region adopted a model that stresses the importance of layperson action in an emergency. This is a paradigm shift from the previous “call 911 and wait” to one of engaging the layperson in providing basic aid after calling for help. Our system started with focus on three key emergent conditions as targets for prehospital citizen intervention: out-of-hospital cardiac arrest (OHCA), opioid overdose, and severe hemorrhage.

To lay a foundation for citizen response, our 911 infrastructure incorporates a bystander notification system, PulsePoint (www.pulsepoint.org). When 911 dispatches units to an OHCA, laypeople within a quarter-mile radius of the scene are simultaneously dispatched via GPS localization. The smartphone-based application also provides access to our county-wide Automated External Defibrillator (AED) registry on the map.

Public safety and government partners worked with local philanthropic and healthcare entities to allow for implementation of PulsePoint and initiate mass-training programs in public arenas such as schools and universities, religious sites, and local events. Where early efforts concentrated on CPR and AED, the citizen response model also began opioid overdose and severe hemorrhage intervention teaching.

Regarding efforts for severe external hemorrhage, the national Stop the Bleed (STB) Campaign empowers laypeople in first response to bleeding emergencies, especially of the extremities. Through the multidisciplinary work of trauma surgery, emergency medicine, Pittsburgh EMS, and local philanthropic support, we trained over 37,000 individuals and distributed over 500 bleeding control kits, and 9,000 tourniquets to police officers. The program also employs mass training programs at schools. STB efforts in Pittsburgh involved the Jewish community, where several synagogues (including Tree of Life) trained and received bleeding control kits prior to the event on October 27, 2018.

## EVENT TIMELINE AND MEDICAL RESPONSE

### Scene Response

Just before 10 AM on the event date, the Allegheny County 911 Center alerted Pittsburgh EMS and police of a possible active shooter at a synagogue in Squirrel Hill. An EM resident, the City EMS Medical Director, and a City Assistant EMS Medical Director responded to a staging area, while the SWAT/TEMS Medical Director rendezvoused with his team.

The Tree of Life congregation recently had STB training and had a fully stocked STB kit near a main entrance of the facility. Unfortunately, wounded civilians on scene were unable to access the kit due to the ongoing danger of the shooter moving through the structure.

Within minutes of the initial alert, an EMS Command Post was created two city blocks from the active shooter event to allow briefing of all physicians and other personnel by the Incident Commander. At Command Post set-up, gunfire existed in the synagogue and it was unclear how many worshipers were in the synagogue for Sabbath services. The synagogue is home to three congregations, multiple classrooms and a basement meeting room.

**Figure 1:**
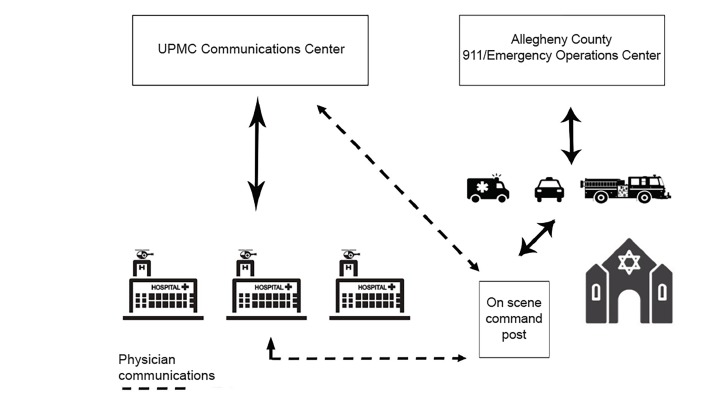
Communications network utilized during the event. *UPMC*, University of Pittsburgh Medical Center.

Meanwhile, staff at the UPMC Communications Center, which provides medical command and hospital notifications for regional ground and aeromedical agencies, along with the county 911/Emergency Operations Center, gathered regional hospital capabilities and relayed that information to the EMS Incident Commander. In addition, communications specialists tracked ambulance transport destinations and provided that information to the on-scene physicians. The EMS physicians on-scene spoke directly to EM leadership, who relayed information to all sites involved and gave real time updates. The on-scene EMS physicians also assisted with patient destination decisions (Figure 1).

TEMS paramedics initiated care of trauma victims based on statewide protocols and at the direction of the physician embedded with the tactical teams. Although there was no shortage of supplies, multiple SWAT operators lacked IFAK and tourniquets when inspected after the event.

Victims, including two civilians and four police officers, were transported to the two closest adult trauma centers. Proximity to the scene was the primary motivation for transporting patients to the closest appropriate facility. Physicians relayed the capacity of the facilities based on real time information from the hospitals. Based on the location of the incident, limited exit routes, and the capacity of the hospitals at the time, most patients were transported to UPMC Presbyterian, which lies on the most direct route from the scene and was prepared to accept a large number of patients. Once captured, the injured gunman was transported to the third adult trauma center; this was a decision made jointly by on-scene physicians and the EMS Incident Commander, seeking to separate the assailant from the victims.

All victims with extremity injuries had a tourniquet(s) placed on-scene or en route. Eleven additional victims in the synagogue were recognized dead and not transported. Prior to leaving the scene, the EMS physicians gathered patient status information from the receiving facilities and this information was relayed to the EMS Chief.

### Timeline of EMS activity on scene

**Figure 2:**
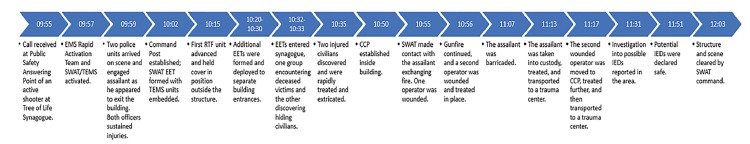
Timeline of EMS activity on scene during the Tree of Life synagogue mass shooting in Pittsburgh, October 27, 2018. *EMS*, Emergency Medical Services; *SWAT*, Special Weapons and Tactics; *TEMS*, Tactical Emergency Medical Services; *EET*, Emergency Entry Team; *RTF*, Rescue Task Force; *CCP*, Casualty Collection Point; *IEDs*, Improvised Explosive Devices.

Note: timeline is based on radio traffic, which may not always reflect real-time (Figure 2).

09:55 – Call received at Public Safety Answering Point of an Active Shooter at Tree of Life Synagogue. Local police patrol units and medic units dispatched to the scene.

09:57 - EMS requested a Rapid Activation Team activation, which includes: One District Chief, five Advanced Life Support units, two Basic Life Support ambulances, two rescue trucks, mass casualty unit, field physician, plus a level-one county MCI response of five ambulances and one supervisor. The SWAT/TEMS team activation also occurred.

09:59 - Two police patrol units arrived and engaged the gunman as he appeared to be leaving the synagogue. Both law enforcement officers incurred injuries during the exchange. EMS requested to have both injured officers brought out to the “warm zone,” a distant safer area with physical structures impeding the shooter’s line of site.

Upon assessment by EMS, one officer was found to have upper and lower extremity wounds (patient 1) that were treated at the scene with compression dressings and a tourniquet before the officer/patient was transferred to a local trauma center. The time from injury to Emergency Department (ED) arrival was under 20 minutes. A second law enforcement officer assessed by EMS had superficial lacerations of the face and was transferred non-emergently to the hospital (patient 2). A summary of patients is presented in Table 2.

10:02 -EMS established an Incident Command post and safe staging area approximately two blocks from the scene. In the next four minutes, five medic units arrived and the EMS District Chief on duty began staging at the post.

Over the next several minutes, arriving SWAT team operators formed an Emergency Entry Team (EET). As TEMS paramedics arrived on scene, they paired with law enforcement to form an RTF. As the EMS Physicians arrived, they reported to the EMS Command post. Local hospitals leaders – using pre-established protocols - were notified of the event to prepare and to gain bed availability data for the EMS incident command post.

10:15 – The RTF unit at the staging post moved closer in to the active area and held a cover position outside the structure.

10:20 to 10:30 – Additional SWAT and TEMS personnel arrived and more EETs formed. These teams deployed to separate entrances of the synagogue. Each EET had embedded TEMS.

10:32 to 10:33 – The EETs entered the synagogue via separate entrances and began clearing their respective areas. One EET remotely assessed their assigned area and found what appeared to be multiple deceased victims. This was confirmed upon entry. The second EET discovered several civilians hiding and escorted them out of the synagogue.

10:35 – Two injured civilians were found as the entry teams and TEMS medics pushed forward; one with a gunshot wound to the lower abdomen, one with an extremity wound (patients 3 and 4). Patient 4 had pressure dressings and a tourniquet applied, followed by extrication from the synagogue.

10:50 – As the EET passed through the structure, members of the TEMS team established a Casualty Collection Point (CCP) inside the building.

10:55 - SWAT contacted the shooter, with initial reports of shots fired and an officer down and officer wounded transmitted. The injured SWAT operator was removed from the area of the gun battle and carried to the CCP established by TEMS (patient 5). At the CCP, the TEMS paramedics and physician assessed the injured officer and identified multiple extremity injuries along with a head wound. The team placed tourniquets on the bleeding extremities and bandaged the head wound. Once extricated, the officer went to a Level I trauma center via ambulance. Time from TEMS contact to ED arrival was approximately 20 minutes.

10:56 - A second SWAT operator (patient 6) suffered an upper extremity wound but was unable to move to the CCP because of his position and the ongoing gunfight. Another SWAT operator applied a tourniquet.

11:07 – The assailant was barricaded.

11:13 – The shooter (patient 7) was taken into custody. His extremity wounds received a tourniquet and hemostatic dressings, and he was taken to the third trauma center.

11:17 – The second wounded SWAT operator (patient 6) moved to the CCP; the team noted an upper extremity wound. Despite initial placement of a tourniquet, hemostasis was inadequate. A second tourniquet and a hemostatic dressing then controlled the bleeding. The wounded SWAT officer walked to an ambulance and arrived at a Level I trauma center approximately 38 minutes after TEMS contact.

11:31 - Reports of possible Improvised Explosive Devices (IEDs) inside the structure and the shooter’s vehicle. All units and individuals staged in the warm zone pulled back.

11:51 - All potential IEDs are inspected by Explosive Ordinance Disposal and declared safe.

12:03 - Structure and scene declared safe and cleared by SWAT command.

### Hospital Response

Pittsburgh has three level-one adult trauma centers: UPMC Presbyterian (2.4 miles from the scene), UPMC Mercy (3.7 miles), and Allegheny General Hospital (8 miles). The only pediatric trauma center is the Children’s Hospital of Pittsburgh of UPMC (2.7 miles). UPMC Presbyterian Hospital, the closest trauma center to the incident, had ten patients in the ED (an atypically low number) when the shooting began. There were two attending physicians and four residents on duty, along with a trauma team staffed by one attending trauma surgeon and four surgical residents.

At 10:04 am, the ED received the first notification of the active shooter situation. ED and trauma staff and the hospital AOD immediately activated the hospital MCI plan. However, due to confusion in terminology between the AOD and hospital operator, a “Bronze Alert,” the hospital’s designation for an active shooter *inside* the building, was sent out through the Emergency Notification System (ENS) to more than 10,000 staff members. This led to confusion amongst some off-duty providers as to whether it was safe to respond to the hospital and amongst those already in the building as to the location of the incident. This represents one area for improvement from the incident.

Nonetheless, many providers quickly mobilized to the ED. In a trend like that noted in past mass shooting events, many of these providers “self-dispatched” from other areas in the hospital and from home. The attending trauma surgeons also communicated internally using a group-text. Within 45 minutes of the initial notification, there were approximately 100 additional providers and ancillary staff ready to receive wounded patients, including physicians from emergency medicine, trauma, vascular, orthopedic, and neurological surgery, anesthesia, and critical care.

During the time that it took to declare the shooting scene safe, information on the number and type of patients being transported varied. Hospital providers, getting information from news media, personal contacts, and official channels, had wide estimates - ranging from four to 40 patients. Ultimately, five patients came to UPMC Presbyterian, one to UPMC Mercy, and one to Allegheny General Hospital.

During and after the incident, dozens of armed law enforcement officers from various agencies (local, county, and state police and federal agents) presented to the ED, leading to some angst with staff about who was responsible for verifying their credentials.

## LESSONS LEARNED FROM THIS EVENT (Table 3)

### Acts of Violence Can Happen Anywhere

Since 2000, Pittsburgh has topped the most livable city lists six times.^5^ The city celebrates its diversity and is known for its friendly demeanor. Pittsburgh is now added to the list of cities that believed “this could never happen here” but experienced an event.

### SWAT Operators Need IFAK Checks, Like for Any Equipment

The SWAT officers tend to have multiple armor sets/configurations that they don and doff. Officers frequently move their IFAK and tourniquets between armor sets. This risks leaving the operators without IFAK and tourniquets because of the need for rapid response and forgetting to transfer their medical gear. We recognized some gaps in this facet that were apparent once debriefing occurred.

### Wounds Can Mirror Modern Combat Theatre Even in Civilian Public Mass Shooting Incidents (CPMS)^6^

The two injured SWAT operators suffered extremity hemorrhage like combat theatre injuries. As with the military, the type of armor used during SWAT operations protects most of the thoracoabdominal area and head but exposes the extremities and face/neck areas to injury.^7^ This makes extremity hemorrhage control extremely important. Civilian wounds from this incident, as observed by on-scene physicians and EMS personnel, closely matched injury patterns in other CPMS events.^8^

### A Single Tourniquet May Not Achieve Adequate Hemostasis

Both SWAT operators wounded in the shooting required multiple tourniquets on their wounded limbs. Each tourniquet was properly placed, tightened, inspected, and re-tightened before deploying a second tourniquet. As noted, one operator suffered upper extremity bleeding that was not adequately controlled by a single tourniquet. Due to his location, he was isolated from TEMS personnel for ~18 minutes due to active gunfire and juxtaposition to the rest of the team. This case illustrates the importance of every officer having at least one tourniquet on their equipment, and having multiple tourniquets and IFAK for select personnel who are “in close” to any exchange of gunfire.

### TEMS Elements Deployed As Far Forward as Safely Possible Save Lives

In the instance of the most critically injured SWAT operator, minutes elapsed between him being shot multiple times and delivery to the TEMS staging area. Immediate point-of-injury care was started by the embedded SWAT physician (the team’s medical director) and a full complement of TEMS medics. This allowed for a full assessment and treatment in under three minutes with transport immediately afterward. Two other patients with potentially life-threatening extremity hemorrhages received TEMS forward care; both patients’ wounds could have easily resulted in death if delays occurred. As such, aggressive uniformed officer engagement with the assailant, persistent and infiltrative tactics by SWAT operators and accompanying TEMS units, and parallel formation and utilization of RTFs should serve as a response model when feasible.

### The Location of Staged Medical Equipment Matters

On scene medical equipment was not accessed because of security concerns. The synagogue’s STB kit was in a visible, central location – a location creating danger since the shooter had access to the area. Although we feel that STB training is an invaluable community resource, we did learn in this case that the location of staged equipment is a critical (and perhaps under-emphasized) point.

### Training on Terminology and Activation of Hospital-wide Alerts is Critical and Requires Frequent Reinforcement

The initial activation of the Bronze alert led to confusion. Our hospitals have responded with increased training for staff and updated guidelines on how to activate the MCI plan.

### In the Event of an MCI, Many Providers Will Self-present to the Hospital Without Being Called

While it is important to have a pre-designated system for calling in back-up, this volunteerism can create the possibility of having too many providers respond. A process should be in place to screen, allocate, and decline use of additional volunteers. Our hospital MCI plan is being updated to establish a “volunteer” center in an area separate from the ED.

### MCI Planning Should be Multi-disciplinary and Involve Both Healthcare Providers and Ancillary Services. Frequent Exercises are Crucial

The hospital response involved a highly coordinated set of actions involving numerous personnel and predesignated supplies. These actions all came from a plan, created in advance and practiced in both table-top and simulation drills.

### Casualty Data are Unreliable Early in an MCI or Active Shooter Situation. Err on the Side of Over-estimating Need During the Initial Response^9^

With numerous sources of information, accurate planning based on need is challenging. A lesson learned from previous drills was that of having MCI plans (prehospital and hospital) that are activated based on known *or* anticipated victims to avoid delay or an inadequate medical response. In this event, the number of potential victims was initially unknown; the closest adult trauma center initiated their MCI plan based on the limited information available, “preparing for the worst.” As a result, the hospital easily accepted all incoming patients and had the capacity to care for more injured patients if needed. Similarly, the other two adult trauma centers enacted response plans, each ready to accept patients above the usual expected for a Saturday morning.

### Have Plans to Confirm the Identity of Law Enforcement Officers and to Manage Their Flow into Care Sites

While understandable (and perhaps unavoidable) that law enforcement will present to the hospital, preparedness efforts should include coordination with hospital security personnel to create plans for their access.

## CONCLUSION

The mass shooting incident at the Tree of Life synagogue on Oct 27, 2018 in Pittsburgh used a coordinated multi-agency response. Planning, practice in advance, close medical provider and safety officer integration, scene safety, initial evaluation zones, and tourniquet use saved lives in this event, and we learned lessons to improve future preparations and responses.

## Figures and Tables

**Table 1:** Contents of Individual First Aid Kits (IFAK) carried by Pittsburgh SWAT officers.

1× - Ratcheting medical tourniquet (RMT) 1.5″ - Tactical
1× - Hemostatic gauze bandage
1× - Compression bandage
1× - Nasopharyngeal airway 28Fr with lubricant
1× - FASTBreathe thoracic seal (FTS) - vented
1× - FAST combat wound seal (CWS)6
1× - Compressed sterile gauze 4″ × 4 yards
2× - Tefla non-adherent pad 3″ × 6″
1× - Emergency Mylar blanket
1× - Band-aid pack
1× - Surgical cloth tape 1″ × 10 yards
1× - HD nitrile gloves (pair) extra large
1× - Activity trauma shears 5″
1× - Casualty/treatment card
1× - Active trauma pouch “ATP” vehicle system

**Table 2:** Description of injuries and on-scene medical interventions.

Patient	Description	On-Scene Interventions
1	Police officer with upper/lower extremity wounds	Tourniquet, compression dressings
2	Police officer with superficial facial lacerations	Simple bandages
3	Civilian with gunshot wound to lower abdomen	None
4	Civilian with extremity injury	Tourniquet and compression dressings
5	SWAT officer with head, neck, multiple extremity wounds	Tourniquets, wound care
6	SWAT officer with extremity wound	Tourniquets, hemostatic agent, pressure dressing
7	Shooter with extremity wounds	Tourniquet, hemostatic agent, pressure dressing
8	SWAT officer with hearing loss	Not transported by EMS

*SWAT*, special weapons and tactics; *EMS*, emergency medical services.

Additionally, eleven victims sustained fatal wounds on-scene of the incident.

**Table 3:** Lessons learned from the Tree of Life synagogue mass shooting in Pittsburgh, October 27, 2018.

Preparation and planning Acts of violence can happen anywhere. The location of staged medical equipment matters. Training on terminology and activation of hospital-wide alerts is critical and requires frequent reinforcement. Mass casualty planning shuold be multi-disciplinary and involve both healthcare providers and ancillary services. Frequent exercises are crucial. Have plans to confirm the identity of law enforcement officers and to manage their flow into care sites. Incident response SWAT operators need IFAK cheks, like for any equipment. Wounds can mirror modern combat theatre even in civilian public mass shooting incidents. A single tourniquet may not achieve adequate hemostasis. TEMS elements deployed as faw forward as safely possible save lives In the event of a mass casualty incident, many providers will self-present to the hospital without being called. Casualty data are unreliable early in an MCI or active shooter situation. Err on the side of over-estimating need during the initial response.

*SWAT*, Special Weapons and Tactics; *IFAK*, Individual First Aid Kit; *TEMS*, Tactical Emergency Medical Services; *MCI*, Mass Casualty Incident.
